# 

**Published:** 1892-11

**Authors:** 


### Ohio State Dental Society.

The next annual meeting of the Ohio State Dental Society will be held at the Neil House, in Columbus, beginning on the first Tuesday of December, 1892. This will be a very desirable place for the meeting; the building has been remodeled and refitted entire, and is under new management, and the best accommodation, in every respect, is assured. A large and commodious hall for the meeting will be furnished free of expense to the society.

The Executive Committee has been unceasing in its efforts to secure a good programme for the occasion, but at the present we are only able to give it in part; quite a number who were expected to read papers and present subjects have not as yet been heard from. The following subjects, however, will be presented, viz:

A paper on "Mechanical Abrasion of the Teeth."

A clinic, "Contouring with Gold and Plastics, Demonstrating the Construction and Use of Matrices and Supports;" also "Immediate Root Filling," by Dr. J. E. Cravens, of Indianapolis.

Second.—"Crown Work (Gold)," by Dr. Grant Mitchell, Canton, O.

Third.—Paper, "Present and Future of Prosthetic Dentistry," by Dr. Geo. H. Wilson, Cleveland, Ohio.

Fourth.—"An Exhibit in Crown and Bridge Work," by Dr. J. M. Cook, Toledo, O.

Fifth.—"A Talk on Some Practical Things in Dentistry," by Dr. J. G. Templeton, Pittsburg, Pa.

A number of other papers will be presented and clinics held, the particulars of which we cannot now give.

The confident expectation is that this will be the largest meeting of this society for many years; and it is to be hoped that the profession of the State will be largely represented, and that no one who has any State pride will let anything, which he can control, prevent his being present. The expectation is that the meeting will continue till Friday afternoon.

### The Pan-American Medical Congress.

The Preliminary Announcement of the Pan-American Medical Congress, to be held at Washington, D. C., U. S. A., September 5th to 8th, inclusive, 1893, is just at hand.

From this Announcement it is fair to conclude that this will be the largest gathering of medical men that has ever been convened in this country.

The initiative steps for its organization were taken at the annual meeting of the American Medical Association, in Washington, May 5th, 1891, when Dr. Charles A. L. Read, of Cincinnati, introduced the following:

*Resolved.* That the American Medical Association hereby extends a cordial invitation to the Medical Profession of the Western Hemisphere, to assemble in the United States in an Inter-Continental American Congress.

*Resolved.* That the Committee on Nominations be, and is hereby instructed to nominate one member for each State and Territory, and one each from the Army, Navy, and Marine Hospital Service, who shall constitute a committee, which is hereby instructed to effect a permanent organization of the proposed Inter-Continental American Medical Congress, and to determine the time and place at which the same shall be held.

These resolutions were unanimously adopted, and the Nominating Committee at once elected.

After this the Congress of the United States by joint resolution authorized the President to invite certain governments to send delegates to the Pan-American Medical Congress. That resolution is as follows:

A joint resolution was unanimously adopted by the Senate, June 3, 1892, concurred in by the House of Representatives, July 14, 1892, and approved, July 18, 1892, by which the President was authorized and requested to invite the several governments of the Western Hemisphere to send official delegates to the meeting of the Pan-American Medical Congress to be held in the city of Washington, September 5, 6, 7, and 8th, Anno Domini 1893.

A series of General Regulations and also Special Regulations and By-laws for the conduct of the Congress were draughted and adopted; these make provision for a very perfect organization.

The following are the constituent countries of the Pan-American Medical Congress: Argentine Republic, Bolivia, Brazil, British North America, British West Indies, (including B. Honduras), Chili, Dominican Republic, Honduras (Sp.), Mexico, Paraguay, Peru, Salvador, Republic of Colombia, Republic of Costa Rico, Equador, Guatemala, Hayti, Kingdom of Hawaii, Spanish West Indies, United States, Uruguay, Venezuela, Danish, Dutch, and French West Indies.

The Sections of the Congress shall be as follows:

1. General Medicine. 2. General Surgery. 3. Military Medicine and Surgery. 4. Obstetrics. 5. Gynæcology and Abdominal Surgery. 6. Therapeutics. 7. Anatomy. 8. Physiology. 4. Diseases of Children. 10. Pathology. 11. Ophthalmology. 12. Laryngology and Rhinology. 13. Otology. 14. Dermatology and Syphilography. 15. General Hygiene and Demography. 16. Marine Hygiene and Quarantine. 17. Orthopædic Surgery. 18. Diseases of the Mind and Nervous System. 19. Oral and Dental Surgery. 20. Medical Pedagogics. 21. Medical Jurisprudence. 22. Railway Surgery.

The officers of each section shall consist of Honorary Presidents who shall be residents of the constituent countries of the Congress; one executive President who shall organize the work of the section, direct its deliberations and deliver an inaugural address at its opening session.

The President of the Congress is Wm. Pepper, M.D., LL.D. of Philadelphia, and seventy-two Vice-Presidents selected from all the countries to be represented. The Secretary General is Chas. A. L. Read, M.D., of Cincinnati, there are nine assistant Secretaries. The Treasurer is Abraham M. Owen, M.D., of Evansville, Ind.

The executive President for each section has been appointed, also a number of Honorary Presidents and Secretaries.

Section nineteen, Oral and Dental Surgery, has been organized with M. H. Fletcher, M.D., D.D.S., of Cincinnati, Ohio, as its executive President, and the following Honorary Presidents:

Dr. Jose Joaquin Aguirre, Santiago, Chili.
Dr. R. R. Andrews, Boston.
Dr. E. A. Baldwin, Chicago.
Dr. George Beers, Montreal, Canada.
Dr. S. B. Brown, Fort Wayne, Ind.
Dr. Emegdio Carillo, City of Mexico, Mexico.
Dr. Wm. Carr, New York, N. Y.
Dr. B. H. Catching, Atlanta.
Dr. Geo. J. Fredericks, New Orleans.
Dr. Ricardo Gordon, Matanzas, Cuba.
Dr. J. H. Hatch, San Francisco.
Dr. A. O. Hunt, Iowa City.
Dr. Louis Jack, Philadelphia.
Dr. H. J. McKellops, St. Louis.
Dr. Francis Peabody, Louisville.
Dr. J. C. Storey, Dallas, Texas.
Dr. J. Taft, Cincinnati.
Dr. J. B. Willmot, Toronto, Canada.

Thirteen Secretaries have been appointed, as follows:

Dr. John S. Marshall, (English-speaking), Chicago, Ill.

Dr. N. Etchepareborda, Tacuari 355, Buenos Ayres, Argentine Republic.

Dr. Wilson, La Paz, Bolivia.

Dr. Benicio De Sa, Rio De Janeiro, U. S. of Brazil.

Dr. Luke Teskey, Toronto, Canada.

Dr. Guillermo Vargas Paredes, Carrera 7, num. 638, Bogota, Republic of Colombia.

Dr. J. Luis Estrada, Guatemala City, Guatemala.

Dr. Geo. Herbert, Wailuku Maui, Hawaii.

Dr. Rafael Rico, Escuela de Med., City of Mexico, Mexico.

Dr. A. Lacayo, Granada, Nicaragua.

Dr. Andres G. Weber, Corrales 1, Havana, Cuba.

Dr. Angel Guerra, Monte Video, Uruguay.

Dr. Ramon Campuzano, (Spanish-speaking), Philadelphia, Penna.

### Board of Examiners.

The Ohio State Board of Dental Examiners will meet at the Chittenden Hotel, Columbus, on the last Tuesday in November, where it is important that all those interested should appear. The Board is desirous of completing its business so far as possible at this time.

Grant Mollyneaux,
Secretary of the Board.

### CONTENTS.

### THE DENTAL REGISTER,

A MONTHLY MAGAZINE DEVOTED TO THE INTERESTS OF THE

**DENTAL PROFESSION.**

*Terms Reduced to* $2.00 *Per Year. Single Copies,* 25 *Cents.*

**EDITED BY PROF. J. TAFT, D.D.S.**

—AND—

**Published by SAM'L A. CROCKER & CO., Ohio Dental and Surgical Depot.**

The volume commences in January and closes in December.

The REGISTER being issued monthly is always fresh, therefore Indispensable to the practitioner who desires to keep posted up with the new inventions and improvements which are daily developed. Neither expense nor effort will be spared to give value and variety to its contents. Articles will be illustrated with well executed engravings whenever they will add to the interest or assist to explanation.

Its extensive circulation and frequency of issue make it one of the BEST DENTAL ADVERTISING MEDIUMS in the United States.

All subscriptions must begin with January or July.

Local or special notices, five lines or less, will be inserted for $3 each insertion; over five lines, 50 cts. per line.

All advertising bills payable in advance, or at furthest, after the issue of the first number containing the advertisement. All transient advertisements to be paid for in advance.

☞CONTRIBUTIONS SOLICITED.

Exclusively Editorial Communications should be addressed to the Editor.

The latest number of the REGISTER may be ordered with or without other goods at any time, and all letters should be addressed to

**SAM'L A. CROCKER & CO., Ohio Dental and Surgical Depot, Cincinnati, O.**

